# Supplementary material for: FERN – a Java framework for stochastic simulation and evaluation of reaction networks
Source: BMC Bioinformatics. 2008 Aug 29;9:356. doi: 10.1186/1471-2105-9-356 (PMC2553347; doi:10.1186/1471-2105-9-356)
Supplement: Additional file 1 — FERN distribution, Version 1.3. This archive contains the FERN source code and binaries as well as documentation and example models in FernML and SBML. [file 1471-2105-9-356-S1.zip › fern/doc/javadoc/fern/network/modification/ModifierNetwork.html]

ModifierNetwork


---


|  |  |  |
| --- | --- | --- |
| **Overview** \| **Package** \| **Class** \| **Use** \| **Tree** \| **Deprecated** \| **Index** \| **Help** | |  |
| **PREV CLASS**   **NEXT CLASS** | **FRAMES**    **NO FRAMES**     **All Classes** |  |
| SUMMARY: NESTED \| FIELD \| CONSTR \| METHOD | DETAIL: FIELD \| CONSTR \| METHOD |  |


---


## fern.network.modification Class ModifierNetwork

```
java.lang.Object
  fern.network.modification.ModifierNetwork
```

**All Implemented Interfaces:**: Network

**Direct Known Subclasses:**: CatalysedNetwork, ExtractSubNetwork, ReversibleNetwork

---

``` public abstract class ModifierNetwork extends Object implements Network ```

Base class for modified networks, which implements the full `Network` interface.
Extending classes should override each method which is
different in the modified network (e.g. in the `ReversibleNetwork` only reactions are
virtually doubled, so each method concerning the species do not change).

Note: An extending class should definitely not copy the whole network but should rather
redirect indices to the original network when possible in order to minimize the
necessary memory.

**Author:**
:   Florian Erhard

---

**Constructor Summary**

`ModifierNetwork(Network originalNet)`
  
          Creates a `ModifierNetwork` from an original network.


| **Method Summary** | |
| --- | --- |
| `AmountManager` | `getAmountManager()`             Gets the `AmountManager` of the original network. |
| `AnnotationManager` | `getAnnotationManager()`             Gets the `AnnotationManager` of the original network |
| `long` | `getInitialAmount(int species)`             Gets the initial amount of the specified molecule species. |
| `String` | `getName()`             Gets the name of the original network |
| `int` | `getNumReactions()`             Gets the number of reaction in the original network. |
| `int` | `getNumSpecies()`             Gets the number of species in the original network. |
| `Network` | `getOriginalNetwork()`             Gets the original network. |
| `Network` | `getParentNetwork()`             Gets the parent network. |
| `int[]` | `getProducts(int reaction)`             Gets the products of a reaction in the original network. |
| `PropensityCalculator` | `getPropensityCalculator()`             Gets the `PropensityCalculator` of the original network. |
| `int[]` | `getReactants(int reaction)`             Gets the reactants of a reaction in the original network. |
| `String` | `getReactionName(int index)`             Gets a string representation of the reactio in the original network. |
| `int` | `getSpeciesByName(String name)`             Gets the index of the species by its name in the original network. |
| `String` | `getSpeciesName(int index)`             Gets the name of the species by index in the original network. |
| `void` | `setInitialAmount(int species, long value)`             Sets the initial amount of the specified molecule species. |

**Methods inherited from class java.lang.Object**

`clone, equals, finalize, getClass, hashCode, notify, notifyAll, toString, wait, wait, wait`

**Constructor Detail**

### ModifierNetwork

```
public ModifierNetwork(Network originalNet)
```

:   Creates a `ModifierNetwork` from an original network.

    **Parameters:**: `originalNet` - the network to modify


**Method Detail**

### getName

```
public String getName()
```

:   Gets the name of the original network

    :   **Specified by:**: `getName` in interface `Network`
    :   **Returns:**: name of the original network.

---


### getAnnotationManager

```
public AnnotationManager getAnnotationManager()
```

:   Gets the `AnnotationManager` of the original network

    :   **Specified by:**: `getAnnotationManager` in interface `Network`
    :   **Returns:**: `AnnotationManager` of the original network

---


### getNumReactions

```
public int getNumReactions()
```

:   Gets the number of reaction in the original network.

    :   **Specified by:**: `getNumReactions` in interface `Network`
    :   **Returns:**: number of reactions in the original network

---


### getNumSpecies

```
public int getNumSpecies()
```

:   Gets the number of species in the original network.

    :   **Specified by:**: `getNumSpecies` in interface `Network`
    :   **Returns:**: number of species in the original network

---


### getProducts

```
public int[] getProducts(int reaction)
```

:   Gets the products of a reaction in the original network.

    :   **Specified by:**: `getProducts` in interface `Network`
    :   **Parameters:**: `reaction` - index of the reaction in the original network **Returns:**: indices of the products in the original network

---


### getReactants

```
public int[] getReactants(int reaction)
```

:   Gets the reactants of a reaction in the original network.

    :   **Specified by:**: `getReactants` in interface `Network`
    :   **Parameters:**: `reaction` - index of the reaction in the original network **Returns:**: indices of the reactants in the original network

---


### getSpeciesName

```
public String getSpeciesName(int index)
```

:   Gets the name of the species by index in the original network.

    :   **Specified by:**: `getSpeciesName` in interface `Network`
    :   **Parameters:**: `index` - index of the species the original network **Returns:**: name of the species

---


### getSpeciesByName

```
public int getSpeciesByName(String name)
```

:   Gets the index of the species by its name in the original network.

    :   **Specified by:**: `getSpeciesByName` in interface `Network`
    :   **Parameters:**: `name` - name of the species **Returns:**: index of the species in the original network

---


### getAmountManager

```
public AmountManager getAmountManager()
```

:   Gets the `AmountManager` of the original network.

    :   **Specified by:**: `getAmountManager` in interface `Network`
    :   **Returns:**: `AmountManager` of the the original network

---


### getPropensityCalculator

```
public PropensityCalculator getPropensityCalculator()
```

:   Gets the `PropensityCalculator` of the original network.

    :   **Specified by:**: `getPropensityCalculator` in interface `Network`
    :   **Returns:**: `PropensityCalculator` of the the original network

---


### getReactionName

```
public String getReactionName(int index)
```

:   Gets a string representation of the reactio in the original network.

    :   **Specified by:**: `getReactionName` in interface `Network`
    :   **Parameters:**: `index` - index of the reaction in the original network **Returns:**: string represenation of the reaction

---


### getInitialAmount

```
public long getInitialAmount(int species)
```

:   **Description copied from interface: `Network`**
:   Gets the initial amount of the specified molecule species.

    :   **Specified by:**: `getInitialAmount` in interface `Network`
    :   **Parameters:**: `species` - index of the species **Returns:**: initial amount of the species

---


### setInitialAmount

```
public void setInitialAmount(int species,
                             long value)
```

:   **Description copied from interface: `Network`**
:   Sets the initial amount of the specified molecule species.

    :   **Specified by:**: `setInitialAmount` in interface `Network`
    :   **Parameters:**: `species` - index of the species: `value` - initial amount of the species

---


### getOriginalNetwork

```
public Network getOriginalNetwork()
```

:   Gets the original network. If there is a higher hierarchy of modified networks
    (e.g. a `ExtractSubNetwork` of a `ReversibleNetwork` of a
    `AutocatalyticNetworkExample`), the highest network (here the `AutocatalyticNetworkExample`
    is returned.

    :   **Returns:**: the original network

---


### getParentNetwork

```
public Network getParentNetwork()
```

:   Gets the parent network. If there is a higher hierarchy of modified networks
    (e.g. a `ExtractSubNetwork` of a `ReversibleNetwork` of a
    `AutocatalyticNetworkExample`), the parental network (here the `ReversibleNetwork`
    is returned.

    :   **Returns:**: the parent network


---


|  |  |  |
| --- | --- | --- |
| **Overview** \| **Package** \| **Class** \| **Use** \| **Tree** \| **Deprecated** \| **Index** \| **Help** | |  |
| **PREV CLASS**   **NEXT CLASS** | **FRAMES**    **NO FRAMES**     **All Classes** |  |
| SUMMARY: NESTED \| FIELD \| CONSTR \| METHOD | DETAIL: FIELD \| CONSTR \| METHOD |  |


---
